# Angiomotin and angiomotin like proteins, their expression and correlation with angiogenesis and clinical outcome in human breast cancer

**DOI:** 10.1186/1471-2407-6-16

**Published:** 2006-01-23

**Authors:** Wen G Jiang, Gareth Watkins, Anthony Douglas-Jones, Lars Holmgren, Robert E Mansel

**Affiliations:** 1 Metastasis and Angiogenesis Research Group, Wales College of Medicine, Cardiff University, Cardiff, UK; 2 Department of Pathology, Wales College of Medicine, Cardiff University, Cardiff, UK; 3 Department of Oncology, Karolinska Institutet, Stockholm, Sweden

## Abstract

**Backgound:**

Angiomotin is a newly discovered molecule that regulates the migration and tubule formation of endothelial cells. It therefore has been implicated in the control of angiogenesis under physiological and pathological conditions. This study examined the expression of angiomotin and its analogues, angiomotin-like 1 (L1) and -like 2 (L2) in breast tumour tissues, and analysed their correlation with angiogenesis and clinical outcomes.

**Methods:**

Human breast tissues (normal n = 32 and tumours n = 120) were used. The levels of expression of angiomotin, L1 and L2 were determined using reverse transcription PCR. Microvessels were stained using antibodies against PECAM, von Willebrand factor (factor 8, or vWF) and VE-cadherin. The transcript levels of angiomotin and its analogues were assessed against the clinical and pathological background, including long term survival (120 months).

**Results:**

Breast cancer tissues expressed significantly higher levels of angiomotin transcript, compared with normal mammary tissues (33.1 ± 11 in normal versus 86.5 ± 13.7 in tumour tissues, p = 0.003). Both L1 and L2 were seen at marginally higher levels in tumour than normal tissues but the difference was not statistically significant. Levels of angiomotin were at significantly higher levels in grade 2 and grade 3 tumours compared with grade 1 (p < 0.01 and p = 0.05 respectively). The levels of angiomotin in tumours from patients who had metastatic disease were also significantly higher than those patients who remained disease free (p = 0.03). Multivariate analysis indicated that angiomotin transcript was an independent prognostic factor (p = 0.031). No significant correlations were seen between angiomotin-L1 and L2 with the clinical outcome. Furthermore, high levels of angiomotin transcript were associated with shorter overall survival (p < 0.05). There was a high degree of correlation between levels of vW factor and that of angiomotin (p < 0.05), but not angiomotin-L1 and angiomotin-L2.

**Conclusion:**

Angiomotin, a putative endothelial motility factor, is highly expressed in human breast tumour tissues and linked to angiogenesis. It links to the aggressive nature of breast tumours and the long term survival of the patients. These data point angiomotin as being a potential therapeutic target.

## Background

Angiogenesis is essential in the development and vascular spread of cancer, by providing nutrients, oxygen, and passages for the departing cancer cells [1-3]. The angiogenic process is regulated by a carefully maintained balance between angiogenic factors and angiogenic inhibitors (angiogenic factors). In cancer, the pro-angiogenic factors frequently gain the 'upper hand', which stimulate vascular endothelial cells to growth, migrate and forming new vascular/capillary tubules. Most angiogenic factors are growth factors that increase the proliferation of vascular endothelial cells. Some factors, however, are strongly involved in the migration and morphogenesis of endothelial cells, such as hepatocyte growth factor. These factors are mainly produced by stromal cells and act via a paracrine pathway. Angiogenic factors, their receptors or molecules specific to vascular endothelial cells have been used to assess angiogenesis. Notable ones include von Willebrand factor (factor-8 or vWF), PE-CAM, VE-Cadherin, VEGF-receptors [4-6].

Angiomotin (AF286598) is a molecule recently identified from its ability to bind to angiostatin using a yeast two hybrid screen [7]. Angiomotin exerts a strong effect in inducing the migration and tubule formation from endothelial cells and promotes angiogenesis. The effect appears to be via its interaction with and subsequent inhibition of angiostatin, an angiogenesis inhibitor.  However, other mechanism(s) may also operate, including possible interaction with integrins. Angiostatin is known to inhibit angiogenesis and metastasis in solid tumours [8]. Angiomotin belongs to a new protein family with which its members share sequence and structural similarities. Two other known members in the family include angiomotin-like-1 and angiomotin-like-2 proteins [9]. Angiomotin-like-1 is also known as junction-enriched and -associated protein (JEAP) that is highly located at tight junctions and co-localised with JEAP [10]. Angiomotin-like-2 has, however, no known functions identified.

The potential pro-motility function of angiomotin has suggested an important role of the molecule in angiogenesis. Indeed, it has been shown that transfection of microcapillary endothelial cells with angiomotin expression vector increases the migration and tubule forming of the cells [7]. Angiomotin deficient mice died in their early days due to a migration defect during their development, further indicating the potential role of angiomotin in cell motility [11]. The important biological role of angiomotin and its analogues indicates that it may play an important role in angiogenesis in tumours. Indeed, angiomotin has been found to be expressed highly in vessels of Kaposis sarcoma, and weakly in vessels from normal tissues in an early study [7]. However, the expression, distribution pattern and the clinical implications of angiomotin in other tumour types are yet to be explored.

Breast cancer is the leading female cancer in U.S. and U.K. The metastatic spread of the tumour is the primary cause of death of the patients. In the past decade, angiogenesis has been shown to be an important biological marker in predicting prognosis and clinical outcome of patients with breast cancer. Traditionally, angiogenesis has been assessed using markers including von Willebrand factor (vWF), VE-cadherin (also known as cadherin-5) and PE-CAM (CD31), and has been found to be increased in tumour tissues compared with normal tissues [12-15]. Micro-vessel density (MVD) has been used as a mean to calculate angiogenesis in these studies and has been shown to be associated with the progress and metastasis of breast cancer [16,17]. A number of the angiogenic factors, such as VEGF has also shown to be linked to prognosis in patients with breast cancer.

We examined the expression of angiomotin and its analogue molecules angiomotin-like-1 and like-2 in a cohort of breast tumours against the clinical information. Here, we report for the first time the aberrant expression of angiomotin in breast tumours, its correlation with angiogenesis and association with metastatic disease in patients with breast cancer.

## Methods

### Materials

RNA extraction kit and RT kit were obtained from AbGene Ltd, Surrey, England, UK. PCR primers were designed using Beacon Designer (Palo Alto, California, USA) and synthesised by Invitrogen Ltd (Pasley, Scotland, UK). Molecular biology grade agarose and DNA ladder were from Invitrogen (Pasley, Scotland, UK. Master mix for routine PCR and quantitative PCR was from AbGene (Surrey, England, UK). Rabbit anti-human VE-cadherin and anti-factor-8 (von Willebrand Factor), goat anti-rabbit and an universal staining kit were from Santa Cruz Biotechnology Ltd (Santa Cruz, CA, USA), and Vector Laboratories (Nottingham, England, UK), respectively.

### Sample collection

Breast cancer tissues (n = 120) and normal background tissues (n = 32) were collected immediately after surgery and stored in the deep freezer until use. Patients were routinely followed clinically after surgery. The median follow-up period was 120 months (June 2004). The presence of tumour cells in the collected tissues was verified by a consultant pathologist, who examined H&E stained frozen sections. Details of histology were obtained from Pathology reports and re-verified by a consultant pathologist (ADJ) (table 1). Patients were routinely followed up on a regular basis and details stored in a database.

### Tissue processing, RNA extraction and cDNA synthesis

Frozen sections of tissues were cut at a thickness of 5–10 μm and were kept for immunohistochemistry and routine histology. Additional 15–20 sections were mixed and homogenised using a hand-held homogeniser, in ice cold RNA extraction solution. The concentration of RNA was determined using a UV spectrophotometer. Reverse transcription was carried using a RT kit with an anchored oligo-dt primer supplied by AbGene, using 1 μg total RNA in 96-well plate. The quality of cDNA was verified using β-actin primers.

### Quantitative analysis of the transcripts of angiomotin, angiomotin-related molecule, and endothelial markers

The level of angiomotin, angiomotin-like-1 and angiomotin-like-2, PECAM-1, VE-cadherin and factor-8 transcripts from the above-prepared cDNA was determined using a real-time quantitative PCR, based on the Amplifluor™ technology, modified from a method previous reported [17,18]. Briefly, pairs of PCR primers were designed using the Beacon Designer software (version 2, California, USA). To one of the primers, an additional sequence, known as the Z sequence (5'actgaacctgaccgtaca'3) which is complementary to the universal Z probe (Intergen Inc., Oxford, England, UK), was added. Sequences of the respective primers were: Angiomotin (5'atacggtgatggagaaacag'3 and 5'ctgaagaactgcgactgtg'3), Angiomotin-like-1 (5'catgagagcctgaccaga'3, and 5'cctcatttcactgtccatct'3), angiomotin-like-2 (5'caccatcaccaccaccat'3 and 5'agaaacagcagcagcagtag'3), PECAM-1 (CD31)(5'ccatcatgggaggtgatg'3 and 5' actgaacctgaccgtacatgctgagacctgcttttc'3), VE-cadherin (5' gggagaccacgcctctgtc'3 and 5' actgaacctgaccgtagaggaggccctgggcatctc'3), vWF (5' ggagagatgggacactaaca'3 and 5' actgaacctgaccgtacagtcatatggacgactgaggt'3)and β-actin: 5'atgatatcgccgcgctcg'3 and 5'cgctcgtgtaggatcttca'3 A Taqman detection kit for β-actin was purchased from Perkin-Elmer. The reaction was carried out using the following: Hot-start Q-master mix (Abgene), 10 pmol of specific forward primer, 1 pmol reverse primer which has the Z sequence, 10 pmol of FAM-tagged probe (Intergen Inc., Oxford, England, UK), and cDNA from approximate 50 ng RNA. The reaction was carried out using IcyclerIQ™ (Bio-Rad) which is equipped with an optic unit that allows real time detection of 96 reactions, using the following condition: 94°C for 12 minutes, 50 cycles of 94°C for 15 seconds, 55°C for 40 seconds and 72°C for 20 seconds. The levels of the transcripts were generated from a standard that was simultaneously amplified with the samples. Cytokeratin-19 (CK19) was used for comparison of cellularity during the analysis and primers for CK19 were 5'-caggtccgaggttactgac-3' and 5'-actgaacctgaccgtacacactttctgccagtgtgtcttc-3' respectively [19,20]. The levels of the angiomotin and the angiomotin like transcripts are shown here as the number of the respective copies and the ratio of angiomotin (or its like proteins) vs cytokeratin 19 (CK19).

### Assessment of micro-vessel density (MVD) using anti-von Willebrand Factor (vWF) and VE-cadherin

The assessment of MVD was as previously reported [21,22]. Frozen sections of breast tumour and background tissue were cut at a thickness of 6 μm using a cryostat. The sections were mounted on super frost plus microscope slides, air dried and then fixed in a mixture of 50% Acetone and 50% methanol. The sections were then placed in "Optimax" wash buffer for 5–10 minutes to rehydrate. Sections were incubated for 20 mins in a 0.6% BSA blocking solution and probed with the primary antibody (1:50 dilution) for 2 hours at room temperature. Following extensive washings, sections were incubated for 30 mins in the secondary biotinylated antibody (Multilink Swine anti- goat/mouse/rabbit immunoglobulin, Dako Inc.). Following washings, Avidin Biotin Complex (Vector Laboratories) was then applied to the sections followed by extensive washings. Diamino benzidine chromogen (Vector Labs) was then added to the sections which were incubated in the dark for 5 mins. Sections were then counter stained in Gill's Haematoxylin and dehydrated in ascending grades of methanol before clearing in xylene and mounting under a cover slip. Microvessels were counted using X20 objectives by 3 independent researchers, as we have recently reported [21].

Statistical analysis was carried out using Mann-Whitney U test and the Kruskal-Wallis test. Survival analysis was carried out using the Kaplan-Meier's and Cox Proportional tests with SPSS12 package.

## Results

### Expression of angiomotins in mammary tissues

**Figure 1:**
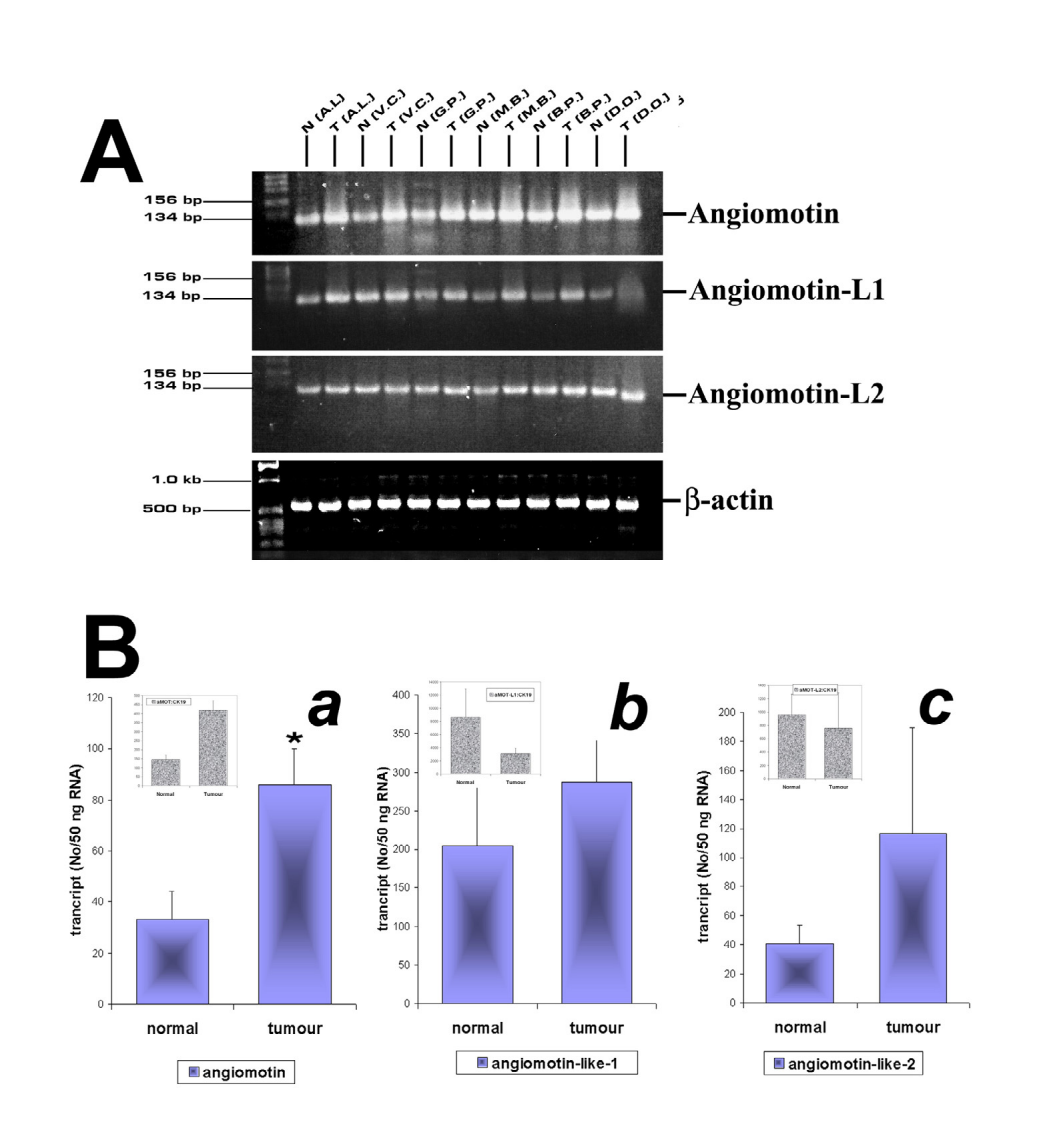
Expression of angiomotin and angiomotin like transcript in normal and tumour tissues using conventional RT-PCR (A) and quantitative real-time PCR (B). A: paired normal (N) and tumour (T) tissues from selected patients. B: levels of the respective transcript and the transcript:CK19 ratio (inserts) from all the samples (normal n = 32 and tumour n = 120). * p < 0.05 vs tumours.

The presence of angiomotin and the angiomotin-like transcripts were detected in both normal and tumour mammary tissues (figure 1A). In these selective paired samples, it was seen that most tumour samples had a stronger angiomotin signal, however, signals for angiomotin-like-1 and -like-2 were not different between normal and tumour tissues.

A quantitative analysis of the molecules indicated that there was a significantly higher level of angiomotin in breast tumour tissues, than in normal tissues (figure 1B-a). The same trend was reflected when the transcripts were normalised by CK19, as measure of controlling the cellularity (figure 1B-a insert). Angiomotin like-1 and like-2 transcript were also high in tumour tissues compared with normal tissues (figure 1B-b and 1B-c, respectively), however, the difference was not statistically significant. Moreover, after being normalised by CK19, as shown in figure 1B inserts, tumour tissues exhibited a lower ratio, compared with normal tissue, although this was not statistically significant. No significant difference between normal and tumour was seen for the angiomotin-like-2:CK19 ratio.

### Angiomotin and histological type and tumour grade

**Figure 2:**
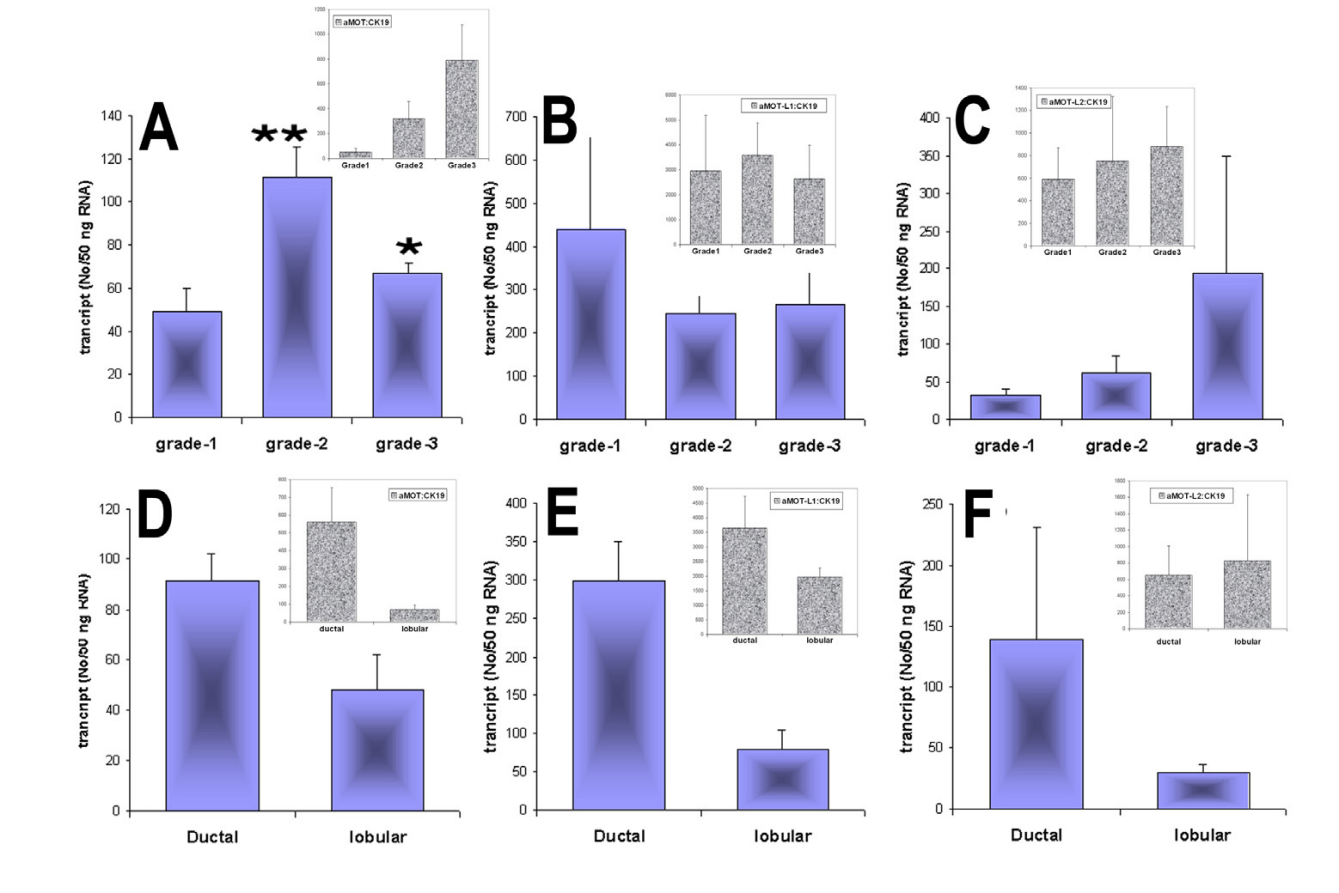
Levels of expression of angiomotins and their relationship with grade (Top panel, A-C) and histological types (bottom panel, D-F). A and D: angiomotin, B and E: angiomotin-like-1, C and F: angiomotin-like-2. ** p = 0.01, * p = 0.049, vs grade 1 tumours. Inserts: respective angiogemotin transcript:CK19 transcript ratio.

Grade-2 and grade-3 tumours had significantly higher levels of angiomotin transcript than grade 1 tumours (p = 0.01 Grade 1 vs grade 2, p < 0.05 grade 1 vs grade 3) (figure 2A). The angiomotin:CK19 ratio was also significantly higher in grade 2 and grade 3 tumours compared with grade 1 tumours (figure 2A insert). No significant difference between different grades was seen with angiomotin-Like-1 or its ratio to CK19 (figure 2B and insert). A marginally high level of angiomotin-like-2 was seen in grade 3 tumours, however neither the transcript nor the transcript:CK19 reached a statistical difference (figure 2C and insert).

Ductal tumours had higher levels of angiomotin (91.4 ± 13.7) than lobular tumours (48.1 ± 16, p = 0.026) (figure 2D). A similar trend was seen with angiomotin-like-1 (298.8 ± 51.4 for ductal and 78.1 ± 25.7 for lobular, p = 0.045). Levels of angiomotin like-2 transcript were marginally higher in ductal tumours (139 ± 92), compared with lobular tumours (29.2 ± 7.3) p = 0.24) (figure 2E and 2F).

### Angiomotin is associated with nodal involvement and predicted clinical outcome

**Figure 3:**
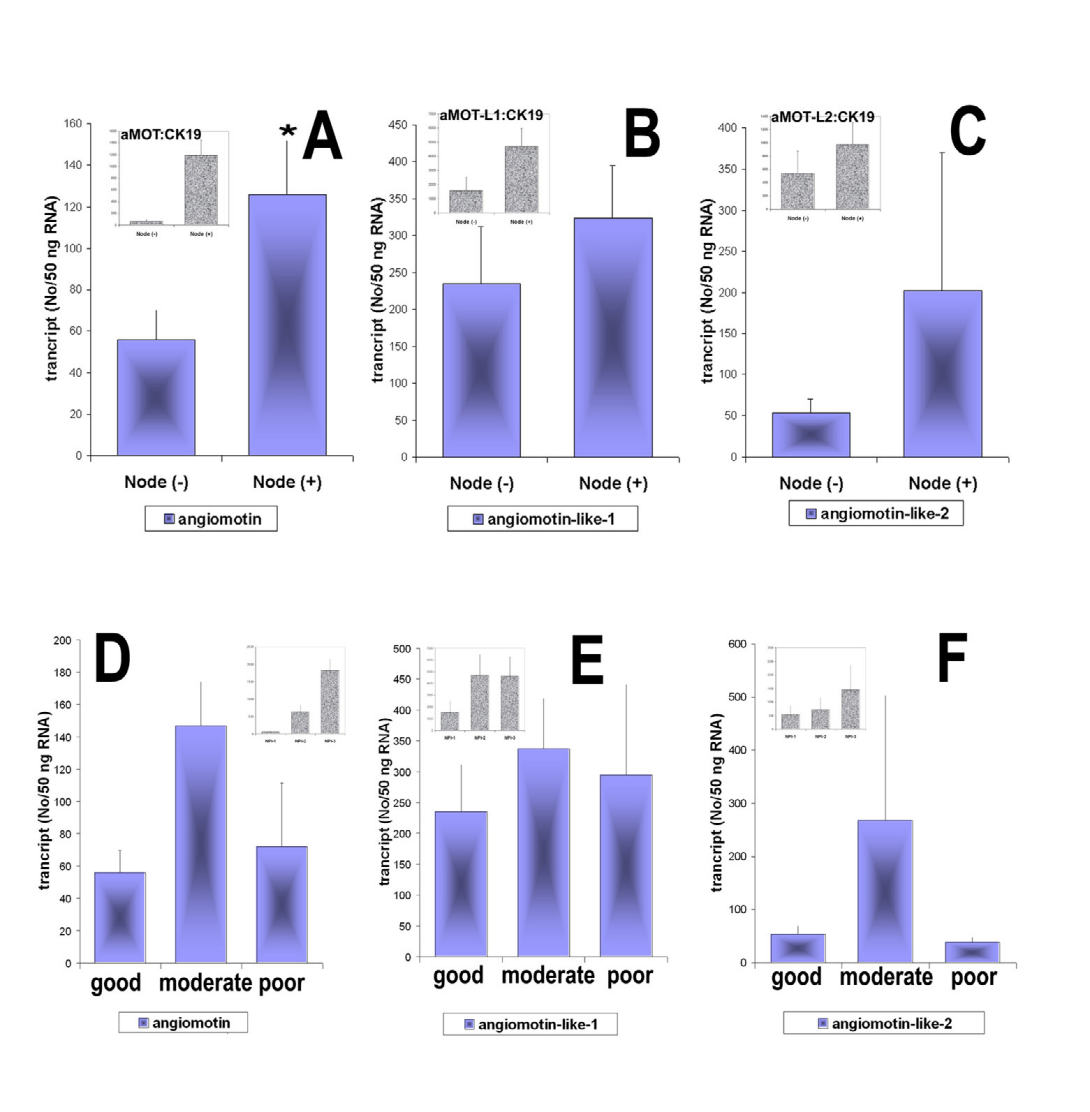
Angiomotins in nodal status (top panel, A-C) and predicted prognosis (bottom panel, E-F). A and D: angiomotin, B and E: angiomotin-like-1, C and F: angiomotin-like-2. A, B AND C : difference between node negative and node positive tumours. * p < 0.01 vs node negative tumours. D, E &F: angiomotins and predicted clinical outcome based on Nottingham Prognostic Index (NPI = (0.2 × size) + grade + Nodal status). NPI<3.4, 3.4–5.4 and >5.4 represented good (15 year survival rate 80%) (NPI1), moderate (15 year survival 42%) (NPI2) and poor prognosis (15 year survival 15%). Inserts: respective angiomotin transcript:CK19 transcript ratio.

There was a significantly higher level of angiomotin and angiomotin:CK19 ratio in tumour with positive axillary nodes (p = 0.0018) (figure 3A and insert). Although there was a trend of higher levels of angiomotin-like-1 and -like-2 in node positive tumours, this was not significant (p = 0.08 and p = 0.6, respectively, figure 3B and 3C).

We have used Nottingham Prognostic Index (NPI) as an indicator for predicted clinical outcome. As shown in figure 3D, although there was a trend of high level of angiomotin transcript in moderate and poor prognostic tumours, statistical difference was only seen with angiomotin:CK19 ratio (figure 3D and its insert). No significant difference was otherwise seen with angiomotin-like-1 and angiomotin-like-2 (figure 3E and 3F).

### Correlation of levels of angiomotin with angiogenesis and other angiogenic markers

We also quantified the levels of VE-cadherin, PECAM-1 and vWF as an indicator of degree of angiogenesis in breast cancer, as we previously reported [21]. There was an increased level of VE-cadherin in breast tumour tissues (2.4 ± 0.64), compared with normal background tissues (1.9 ± 0.61), p = 0.7. Similarly, a high level of PECAM1 (CD31) in tumour tissues (275.0 ± 73.7), compared with normal background tissues (145.7 ± 30.0), p = 0.8. A Spearman correlation test between PECAM1, VE-cadherin and angiomotins revealed a significant correlation between angiomotin transcript and VE-cadherin as well as PECAM1 (r = 0.35 and r = 0.338, p < 0.05 respectively). Angiomotin transcript levels were also significantly correlated with microvessel count (MVD) using anti-vWF (r = 0.400, p < 0.05). There was no significant correlation between levels of these factors and angiomotin-like-1 and angiomotin-like-2.

### Implications of angiomotin with clinical outcome

**Figure 4:**
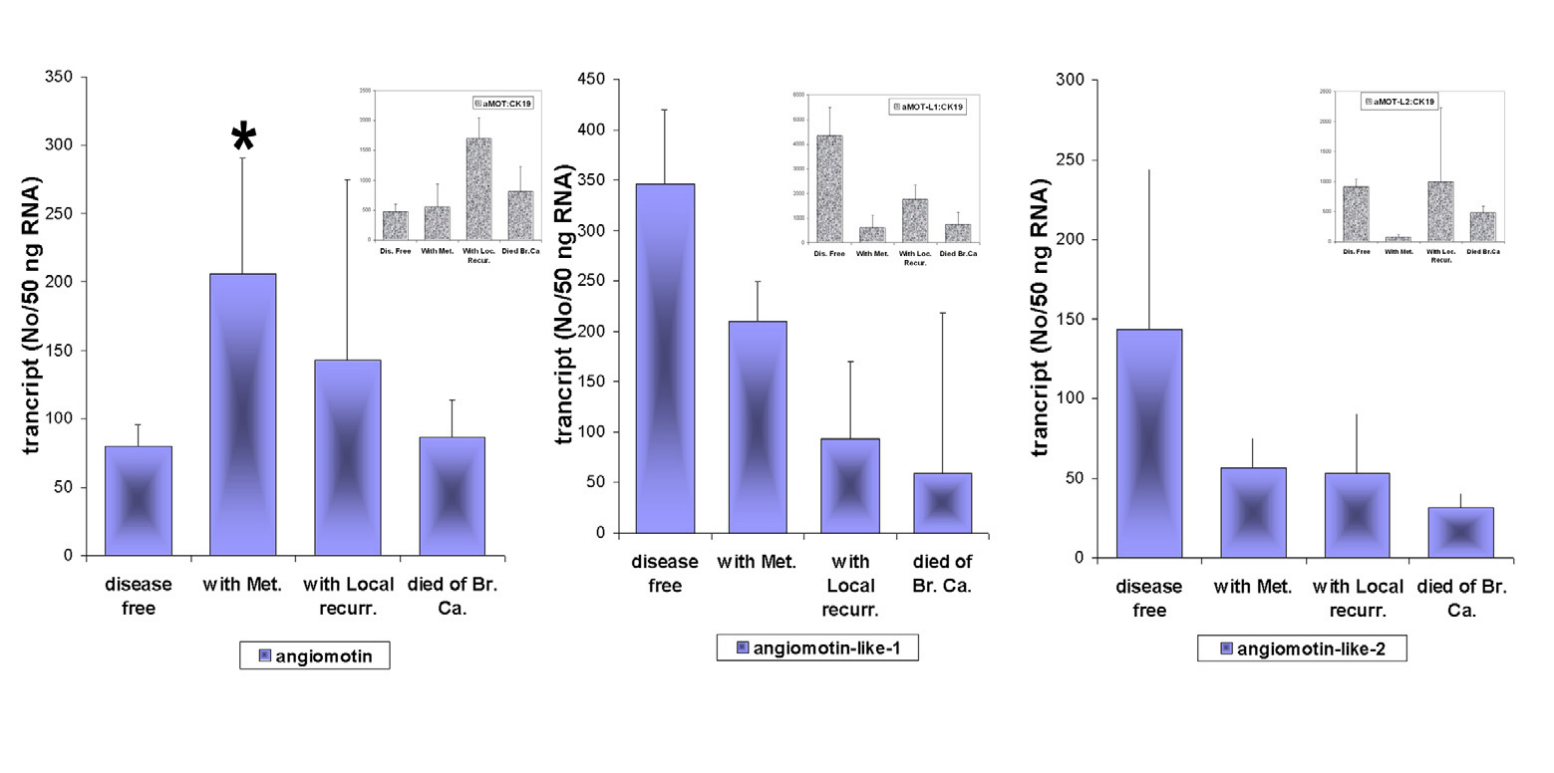
Angiomotins and clinical outcomes. Patients were assessed based on the outcome after 10 years from initial surgery, and were divided into those who remained disease free, with metastasis, with local recurrence and who died of breast cancer related causes. Inserts: respective angiomotin transcript:CK19 transcript ratio. * p = 0.03 vs disease free group.

Following a median 120 month followup, patients were divided into the following groups: those who remained disease free, who developed metastasis or local recurrence, and those who died of breast cancer related disease (excluding non-cancer related deaths). Angiomotin was seen at significantly higher levels in patients who developed metastatic disease compared with those who remained disease free (p = 0.03) (figure 4 left). Although tumours from patients with local recurrence and who died of breast cancer also had a higher level of angiomotin, the difference was nonetheless not significant. Levels of both angiomotin like-1 and like-2 transcripts were lower in patients who had a poor clinical outcome, although the difference was not statistically significant (figure 4 middle and right).

**Figure 5:**
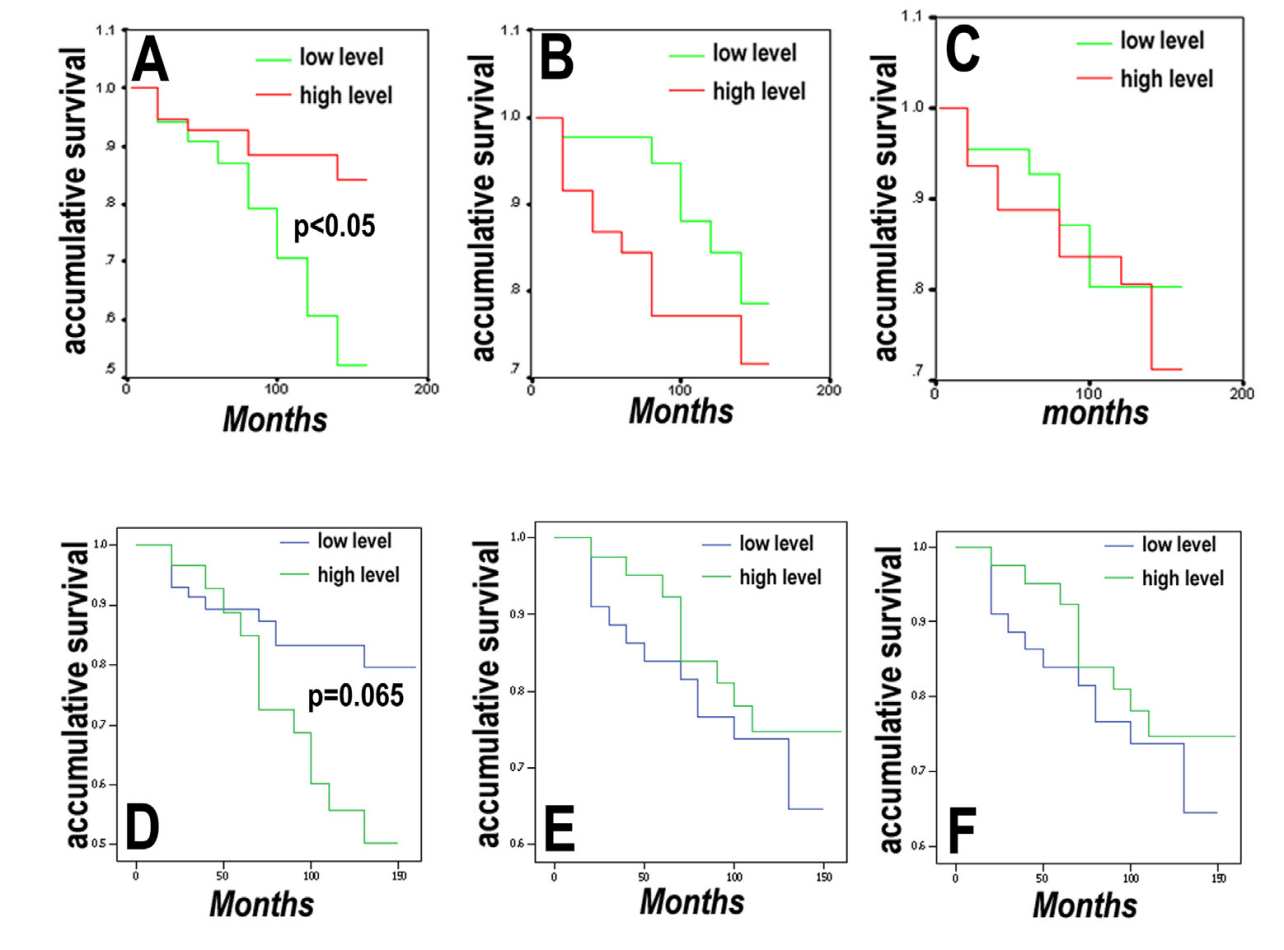
The correlation between Angiomotin (A and D), angiomotin-like-1 (B and E) and angiomotin-like-2 (C and F) and Overall Survival (A-C) and disease free survival (D-F), using Kaplan-Meier survival analysis. Levels of angiomotin transcript were associated with the overall survival (A).

We have divided patients into two groups, those with high levels of angiomotin and those with low levels of angiomotin, by using the prognostic index, NPI, as a general reference. Where a tumour had angiomotin level higher than the mean level of NPI-2 group (NPI 3.4–5.4, with moderate prognosis), it was assigned as high. Kaplan-Meier survival analysis has shown that patients bearing tumours with high levels of angiomotin were associated with a shorter overall survival (119.9 (105.2–134.7 95% CI) months vs 136.5 (124.5–148.4, 95%CI) months for patients with low angiomotin transcript, p < 0.05) (figure 5A). No significant correlation was seen between overall survival and Angiomotin-L1 and L2 (figure 5B and 5C). Similarly, patients bearing tumours with high levels of angiomotin were associated with a shorter disease free survival (108.6 (92.3–125.0 95% CI) months vs 134.3 (122.0–146.7, 95%CI) months for patients with low angiomotin transcript, however, the difference was not statistically significant, p = 0.0634) (figure 5D). No significant correlation was seen between disease free survival and Angiomotin-L1 and L2 (figures 5E and 5F). Using multivariate analysis of the following factors, nodal status, tumour grade, angiomotin, angiomotin-like-1 and angiomotin-like-2, we have found that nodal status (p = 0.0185) and angiomotin transcript (p = 0.031) were independent survival factors.

Furthermore, we have found that angiomotin-L1 was expressed at a significantly lower level in ER positive tumours compared with ER negative tumours (table 2). No significant difference was seen with angiomotin, angiomotin-L2 and ER status.

## Discussion

Angiogenesis is the essential process in the development and spread of breast cancer, by providing blood supply to tumours and escape route for tumour cells to other part of the body. Here we report that angiomotin, a protein that regulate the motility and morphology of endothelial cells is highly expressed in human breast tissues and that its levels are associated with other angiogenic markers and with the clinical outcome in patients with breast cancer.

Angiomotin is a motility regulator of vascular endothelial cells and may be a molecule that links to breast cancer growth and spread by way of stimulating angiogenesis. The current study provides lines of evidence to support this possibility. First, angiomotin is highly expressed in aggressive tumours (grade 2 and 3 and tumours with nodal involvement) than in less aggressive tumours. Second, levels of angiomotin are correlated with levels of angiogenic markers. Third, significantly higher levels of angiomotin transcript are seen in patients with metastatic disease. These data, together with the report that angiomotin directly enhances angiogenesis in vitro and in vivo, suggest that angiomotin is linked to the angiogenic and aggressive nature of breast cancer. However, it would require additional work to verify if angiomotin and angiomotin like proteins can be reliable surrogate markers for angiogenesis.

Although angiomotin-like-1 and -like-2 proteins are also expressed at higher levels in breast tumour tissues, the difference is not significant. There are no consistent patterns to suggest these two analogues of angiomotin are also linked to the aggressiveness of breast tumours, although both of the angiomotin related proteins are expressed in endothelial cells [23]. In fact, the levels of both analogues decrease in tumours that are associated with metastasis and mortality, which is in clear contrast with that of angiomotin. Currently there is no clear explanation to the seemingly different role between angiomotin and angiomotin like proteins. However, angiomotin-like-1 protein is known to be a tight junction related molecule and is highly located in tight junctions [10]. Recent years have seen significant advances in the understanding of the biology and role of tight junction in endothelium as defence mechanism in preventing blood borne cancer cells to escape. Tight junctions in endothelial cells may act as a 'sealing' structure to separate the blood circulation from tissue space [24]. Endothelial tight junctions act as a natural barrier for the vascular spread of cancer cell, by keeping the circulating cancer cells 'at bay-in the circulation'. In epithelial cells, as well as being a permeability barrier, tight junctions act as a strong cell adhesion mechanism and have a potential tumour suppressor role [25]. It has been reported that loss of certain tight junctional molecules, such as ZO-1, ZO-2, and occludin are frequently seen in clinical tumours and the loss of these TJ molecules is associated with the aggressiveness of tumours [26-29]. Thus, it is anticipated that angiomotin-like-1 protein, may act very different from angiomotin, potentially through their participation in tight junctions. Clearly, this is a fertile area to investigate in the future. The study has also indicated that ER negative tumours had a significantly higher level of angiomotin-L1 transcripts than in ER positive tumours. Although angiomotin-L2 transcripts were higher in ER negative tumours than in ER positive tumours, the difference was not statistically significant. In contrast, no difference was seen with angiomotin transcript between the two groups. This presents an interesting link between ER status and the angiomotin-L1 transcript. It has been established that ER negative tumours are associated with a poorer prognosis than ER positive tumours. It is possible that high levels of angiomotin-L1 in the ER negative tumours contribute to this clinical link. Another potential link is that an intrinsic relationship between the expression of angiomotin-L1 and oestrogen in breast cancer may exist. Clearly, more experimental work is required here.

## Conclusion

Although it is at early stage in the investigation of angiomotin and its family into breast cancer, the current study and recent reports have clearly shown the important role of angiomotin in angiogenesis and in the aggressive nature of breast tumours. These data suggest that angiomotin is not only a highly useful prognostic indicator in breast cancer, it may also be a valid therapeutic target in cancer.

## Competing interests

The author(s) declare that they have no competing interests.

## Authors' contributions

WGJ was responsible for study design, data analysis, and preparation of MS, ADJ for pathological information and verification, GW for immunohistochemical work and image analysis, LH for study design and MS preparation, REM for clinical follow-up.

## Pre-publication history

The pre-publication history for this paper can be accessed here:



## Figures and Tables

**Table 1:** Clinical and pathological information of the study cohort. Shown are number of samples in each group

Node status	Node negative	Node positive				
n=	65	55				
Grade	Grade 1	Grade 2	Grade 3			
n=	23	41	56			
Histology	Ductal	Lobular	Medullary	Tubular	Mucinous	Others
n=	94	14	2	2	4	4
TNM staging	TNM 1	TNM 2	TNM 3	TNM 4		
n=	69	40	7	4		
Clinical outcome	Disease free	With Metastasis	With local recur.	Died of breast Cancer	Died of unrelated diseases	
n=	81	7	5	20	7	

**Table 2:** Levels of angiomotin transcript and their relationship with ER status. Shown are number of the respective transcript.

	ER(-)	ER(+)	P value
Angiomotin	87.2 ± 16.8	101.7 ± 29.7	P = 0.67
Angiomotin-L1	383.7 ± 86.0	167.6 ± 39.1	P = 0.024
Angiomotin-L2	174.0 ± 120	31.7 ± 8.6	P = 0.24
